# The genetic architecture of low-temperature adaptation in the wine yeast *Saccharomyces cerevisiae*

**DOI:** 10.1186/s12864-017-3572-2

**Published:** 2017-02-14

**Authors:** Estéfani García-Ríos, Miguel Morard, Leopold Parts, Gianni Liti, José M. Guillamón

**Affiliations:** 1 Departamento de Biotecnología de los alimentos, Instituto de Agroquímica y Tecnología de los Alimentos (CSIC), Avda. Agustín Escardino, 7, E-46980-Paterna, Valencia, Spain; 2 Departament de Genètica, Facultat de Ciències Biològiques, Universitat de València, Dr. Moliner, 50, E-46100 Burjassot, València, Spain; 3 European Molecular Biology Laboratory, Meyerhofstrasse 1, Heidelberg, 69117 Germany; 4 Institute of Research on Cancer and Ageing of Nice (IRCAN), CNRS UMR 7284-INSERM U1081, Faculté de Médecine, Université de Nice Sophia Antipolis, Nice, France; 5 Wellcome Trust Sanger Institute, Hinxton, CB101SA UK

**Keywords:** Quantitative trait loci, Cold adaptation, Industrial yeast, Subtelomeres, Lipid asymmetry, Reciprocal hemizygosity analysis

## Abstract

**Background:**

Low-temperature growth and fermentation of wine yeast can enhance wine aroma and make them highly desirable traits for the industry. Elucidating response to cold in *Saccharomyces cerevisiae* is, therefore, of paramount importance to select or genetically improve new wine strains. As most enological traits of industrial importance in yeasts, adaptation to low temperature is a polygenic trait regulated by many interacting loci.

**Results:**

In order to unravel the genetic determinants of low-temperature fermentation, we mapped quantitative trait loci (QTLs) by bulk segregant analyses in the F13 offspring of two *Saccharomyces cerevisiae* industrial strains with divergent performance at low temperature. We detected four genomic regions involved in the adaptation at low temperature, three of them located in the subtelomeric regions (chromosomes XIII, XV and XVI) and one in the chromosome XIV. The QTL analysis revealed that subtelomeric regions play a key role in defining individual variation, which emphasizes the importance of these regions’ adaptive nature.

**Conclusions:**

The reciprocal hemizygosity analysis (RHA), run to validate the genes involved in low-temperature fermentation, showed that genetic variation in mitochondrial proteins, maintenance of correct asymmetry and distribution of phospholipid in the plasma membrane are key determinants of low-temperature adaptation.

**Electronic supplementary material:**

The online version of this article (doi:10.1186/s12864-017-3572-2) contains supplementary material, which is available to authorized users.

## Background

Low temperature is one of the most important environmental stresses that influences the life and distribution of living organisms. In the yeast *Saccharomyces cerevisiae*, reductions in environmental temperature have widespread effects on growth and survival. At low, but permissive, temperatures (10–18 °C), metabolic activity and growth rates lower. This is relevant for the industrial exploitation of yeast since brewing and some wine fermentations take place at around 12–15 °C. Low temperatures are used in wine fermentations to enhance production and to retain flavor volatiles, which enable the production of white and “rosé” wines with greater aromatic complexity [1, 2].

Yeast undergoes considerable stress during wine fermentation from high concentrations of sugars in grape must, which leads to high osmotic pressure at the beginning of the process. As fermentation proceeds, ethanol accumulation, limiting nitrogen concentration, or even the presence of SO_2_, impose further pressure. In addition to these inherent difficulties to the process, temperatures below the optimum range of growth (around 32 °C) [3] affect yeast growth and fermentation rates, and give rise to both a prolonged lag phase and the production of stuck and sluggish fermentations [4].

Changes in gene expression levels, ploidy and copy number variation (CNV) serve as the main genetic adaptive signatures of wine yeasts to fermentative processes [5]. Other enological traits, such as ethanol production, residual sugar after fermentation, nitrogen uptake and volatile acidity, are complex traits determined by multiple quantitative trait loci (QTL) [6]. The genetic mechanisms that underlie their variation can be identified by a linkage analysis. This approach uses crosses between two phenotypically different strains, and searches for a statistical link between the phenotype and genetic markers of segregant strains [7, 8]. QTL mapping has been successfully applied to high-temperature growth [9–11], sporulation [12–15], cell morphology [16], drug sensitivity [17], ethanol tolerance and growth [15, 18, 19], protein abundance [20, 21], and flocculation [22, 23]. QTL approaches have also been used to dissect the molecular basis of several wine yeast metabolic traits, such as acetic acid production, hydrogen sulfide production, and for the release of volatile phenol [24], nitrogen utilization and metabolite production [25], and the production of acetic acid, glycerol, and residual sugar concentrations [26].

To identify the genetic variants that affect low-temperature adaptation in wine yeast, we performed QTL mapping using a set of segregants derived from a cross between two industrial wine yeast strains with a divergent phenotype at low temperature [27], but with a very similar genotype. We identified four genomic regions located in the different chromosomes implicated in the fermentation process at low temperature in industrial wine yeast, and ran a reciprocal hemizygosity (RH) analysis for validation purposes. We identified subtelomeres to be an important source of genetic variation in industrial yeast [28], and found both the mitochondria and proteins involved in maintaining correct asymmetry and distribution of phospholipid in the plasma membrane played a crucial role in low-temperature growth.

## Results

### Genetic characterization of parental strains

**Fig. 1:**
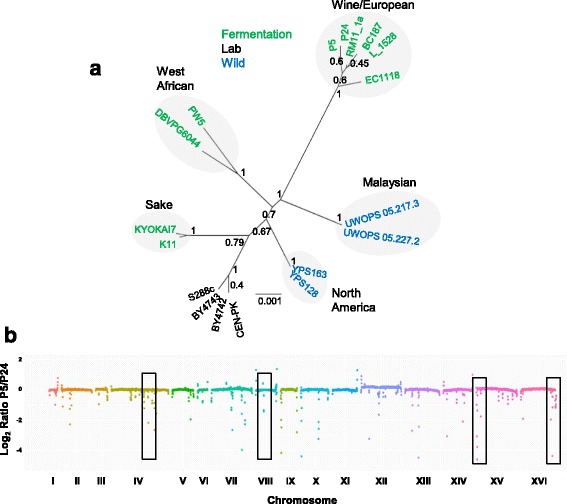
Genomic and phylogenetic analyses of parental strains. **a** The evolutionary history was inferred using the ML method with 1000 replicates of the bootstrap test based on concatenated nucleotide sequences of seven phylogenetic informative loci. Different *colors* denote the strain’s origin. **b** Copy number variation across the genome (chromosomes along x-axis and log2 ratio in y-axis). The four regions with a CNV bigger than 4 kb are marked in the figure

We investigated the genetic basis of low-temperature adaptation in wine yeast in two *S. cerevisiae* enological strains, P5 and P24, characterized in a previous study [27], as displaying extreme differences in fermentation ability at 15 °C. P5 corresponds to commercial strain Lalvin®ICVGRE, which is marketed for temperature fermentations ranging from 15 to 30 °C. P24 has no commercial name since it is undergoing its development stage. The genomes of these two wine strains were sequenced and compared with that of reference strain S288c. We used seven informative genes to classify the parental strain [29] to construct a maximum likelihood phylogenetic tree of 15 strains of different origins. Figure 1a shows the five clean lineage described by Liti et al. (2009) [30], which are well clustered. As expected, both parental strains belonged to the Wine/European lineage with a bootstrap value of 1.

Based on the raw sequence data, 43666 and 42983 mutations in strain P5 and strain P24, respectively, were identified compared to the reference strain. Of the total number of single-nucleotide polymorphism (SNPs), 36830 were common to both wine strains (Additional file 1: Table S1), with 6836 and 6153 specific mutations in P5 and P24 respectively, of which 86 and 82% were homozygous and representing a sequence divergence of 0.05% (Additional file 2: Figure S1). Approximately 30% of the unique SNPs of both wine strains were nonsynonymous changes in the coding region, which resulted in an amino acid change. This set of strain specific mutations was used for the linkage analysis.

Certain classes of variants, such as insertions and deletions (indels), are expected to have dramatic consequences for gene products, and therefore constitute particularly interesting candidates for contributing to phenotypic variation. The comparison with the reference strain yielded 867 and 874 strain specific indels, in which approximately 14 and 13% were within the coding sequence, in the P5 and P24 respectively. When examining the distribution of these strain specific indels to each strain along chromosomes, some (chromosomes X, XI, XV and XVI) showed an enrichment of variants. Within open reading frames (ORFs), the indels with lengths that were multiples of three were highly enriched when compared with the noncoding sequence. This is consistent with strong purifying selection against frameshifts. Those indels in the coding sequence with lengths that were not multiples of three were located mainly in functionally uncharacterized genes. This confirmed that this group of genes was, on average, under lower purifying selection pressure [31].

We also detected the copy number variation of the genomic regions between both strains (CNV). Ninety-three CNV longer than 900 bp were detected across the genome comparison made between both strains. Figure 1b shows the copy number variation (CNV) across chromosomes. Although there were some regions with variation, only four were larger than 4 kb. These four regions were located in chromosome IV and in the subtelomeric regions of chromosomes VIII, XV and XVI. Most of these CNV were classified as transposable elements and subtelomeric regions. These results are in line with previous observations [31], which found very limited CNV in nonsubtelomeric regions and extensive variation in subtelomeric regions.

### Genetic and phenotypic characteristics of segregants

**Fig. 2:**
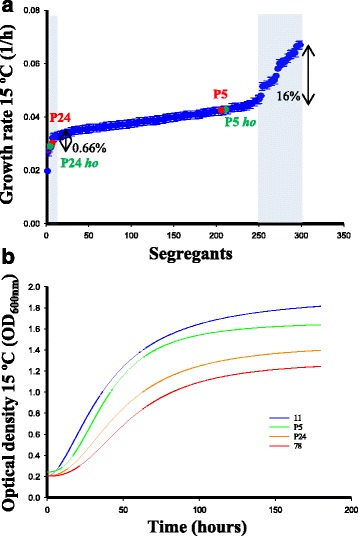
Growth rate variation at low temperature. **a** Growth rate values at 15 °C are shown on the y-axis for 300 ranked segregants. *Blue* indicates transgressive phenotypic space, and segregants with transgressive phenotypes (exceeding two parental standard deviations). *Red dots* indicate parental strains. **b** Detailed growth curves of parental and transgressive segregants (11 and 77) with extreme traits in the P5×P24 cross

For the QTL analysis, we generated populations of segregants by crossing the two wine strains of different cold tolerance for up to 13 generations (F13). This strategy allowed us to increase the resolution by reducing linkage between nearby QTLs. To study the genetic diversity of segregant populations, five differential SNPs of chromosome III in 30 segregants of F6 were genotyped. A recombination frequency of 0.39 was assessed, and there was an average of 10 haplotypes per SNP (data not shown). The F13 segregants were screened for their low temperature adaptation by calculating the maximum growth rate of each segregant and the two parental strains (Fig. 2a).

Transgression levels, that is, the percentage of segregants that exceed the phenotypic range of their parents by at least 2 standard deviations [24], can provide insight into the genetics that underlies complex traits. Sixteen percent of transgressive segregants better performed if compared with the superior parental, while just 0.66% of segregants presented a lower transgressive value than the inferior strain. Trait heritability was over 65%, which indicates the importance of genetic determinism under our conditions. An example of the growth curves of two transgressive segregants is offered in Fig. 2b.

### Low-temperature selection of segregant populations

**Fig. 3:**
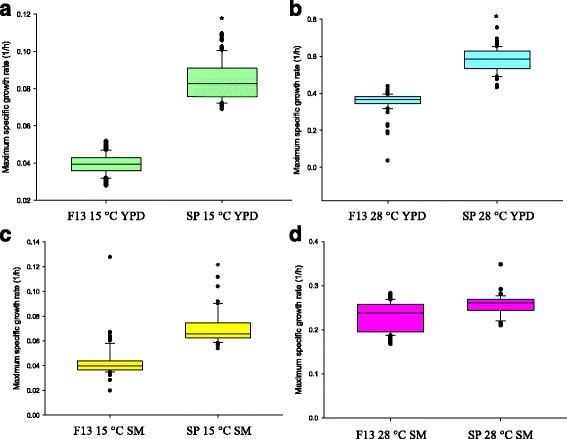
Phenotyping (growth rate) of the hybrid population after the selection experiment compared with the unselected F13 population. Selected population (SP) in the YPD medium (**a**) and synthetic must (SM) (**c**) at 15 °C. Selected population (SP) in YPD (**b**) and SM (**d**) at 28 °C. *Box plot* represents μmax distribution in each population and the *black bar* inside the box represents the mean value. *Significant differences in the SP compared with the F13

The F13 diploid population was subjected to a selection experiment at 15 and 28 °C for approximately 50 generations (8 rounds of batch serial dilution; Additional file 3: Figure S2). Figure 3 shows the improvement in the growth rate (h^−1^) of the selected population (SP) compared with the F13 population. At low temperature, the growth rate of the selected population improved in both yeast extract peptone dextrose (YPD) (97%, Fig. 3a) and synthetic must (SM) (66%, Fig. 3c). The control population, which was cultured at 28 °C, was also phenotyped in both media. The YPD selected population at 28 °C showed an improved growth rate (57%, Fig. 3b). No significant differences were obtained for the SM selected population at 28 °C (Fig. 3d).

To study if the improved growth rate in populations was due to either the temperature selective pressure or an unselective stress, each set was exposed to the opposite temperature to which they were selected. This meant that the SP at 15 °C was grown at 28 °C and that selected at 28 °C was grown at 15 °C, and their growth rates were also compared with the F13 population (Additional file 4: Figure S3). As expected, in YPD medium, the selected population either at 28 or 15 °C didn’t improve its growth or grew worse when was cultivated to the opposite temperature, revealing a selection based on the temperature. However, the selected population in SM also improved its growth when was cultivated in a non-selective temperature, indicating that this complex medium also exerted a strong pressure during the selection.

### Allele frequency analysis reveals that four QTLs are related to low-temperature adaptation during alcoholic fermentation

**Fig. 4:**
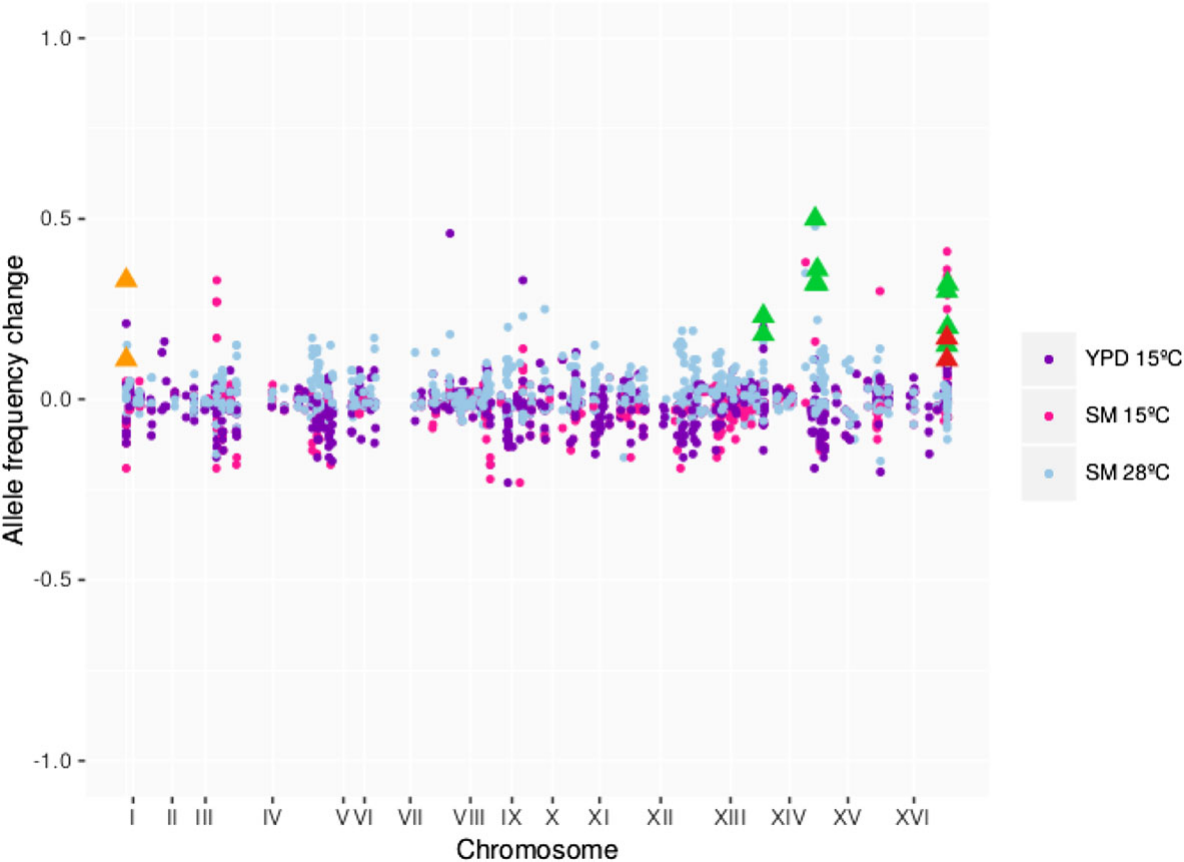
QTL analysis for low-temperature adaptation. Genomic DNA samples were extracted from an unselected pool (F13) and three pools of segregants were selected at 15 °C (YPD and SM) and 28 °C (SM). The figure shows the allele frequency change of the three selected pools compared with the unselected population. QTLs are indicated at the corresponding positions with *orange triangles* (28 °C SM), *red triangles* (15 °C YPD) and *green triangles* (15 °C SM)

A pool of the selected populations at 15 °C in YPD and SM and at 28 °C in SM were whole-genome sequenced and compared with the whole-genome sequence of a pool of the F13 population before selective growth at both temperatures. The allele frequency analysis allowed us to map the QTL intervals responsible for phenotypic variation. Four distinct QTLs for the three assayed conditions (15 °C in YPD, 15 °C in SM and 28 °C in SM) were found (Fig. 4 and Additional file 5: Figure S4). QTLs were detected for 28 °C (orange triangles) on chr I, for 15 °C in YPD (red triangles) on chr XVI and for 15 °C in SM (green triangles) on chr XIII, XIV, XV and XVI. The QTL found at low temperature in YPD overlapped with that located in chromosome XVI at low temperature in SM. This finding suggested that this region could be important for low-temperature adaptation, and independently of media. Except for the QTL located in chromosome XIV, all the others were located in the subtelomeric regions. These results reinforce the hypothesis that subtelomeric regions are a major source of the divergence of genome sequences and gene content in *S. cerevisiae,* and directly contribute to strain-specific adaptation processes.

### Validation of the QTLs detected in subtelomeric regions by reciprocal hemizygosity (RH) analyses

**Fig. 5:**
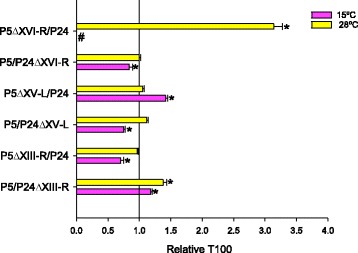
RH analysis of the QTLs detected in subtelomeric regions. Fermentation kinetics of the hemizygous P5/P24 diploid hybrids retained a single copy of the subtelomere (XIII-R, XV-L and XVI-R) either from the superior (P5) or inferior parent (P24), while the other copy was deleted. The fermentation activity of the hemizygous strains is presented as the relative T100 compared with hybrid P5/P24. The T100 value was compared with the control strain normalized as value 1. The T100 of the control strain was: 135 and 755 h at 28 and 15 °C respectively. *Significant differences compared with the control at the same temperature. # Indicates stuck fermentation before T100

Subtelomeric regions are difficult to sequence and assemble due to their wide variation, duplication levels and shared homologies between different chromosome ends. Thus subtelomeres are generally incomplete in most genome projects, which precludes the identification of causative genes of a trait in these regions [32]. To validate the involvement of subtelomeric regions as QTLs of low-temperature adaptation, two hemizygous diploid P5/P24 hybrid strains were constructed, which retained a single copy of the subtelomere from either the superior (P5) or inferior parent (P24), while the other copy was deleted. As growth rate and fermentation activity are directly correlated in these strains [27], they were tested during low-temperature fermentations to estimate the phenotypic differences between the two reciprocal hemizygotes of each subtelomere. Fermentation activity was estimated by calculating the time required to ferment 100% (T100) of the sugars in the SM at 15 and 28 °C and it also is an interesting trait for our aim of detecting genome regions involved in adaptation to low temperature fermentations. The fermentation activity of the hemizygous strains is presented in Fig. 5 as the relative T100 compared with hybrid P5/P24. The parental origin of the subtelomere produced an opposite impact on fermentation activity in most cases. Thus the absence of the P5 right XVI-subtelomere caused a long delay of the end of fermentation at 28 °C and the inability to finish it at a low temperature. Conversely, lack of the same region that belonged to P24 significantly (*p*-value ≤0.05) improved fermentation at low temperature compared with the control hybrid strain P5/P24. Thus the P5 XVI-subtelomere must contain genes of paramount importance for fermentation activity in this strain, regardless of the temperature applied in the process. However, this same region in the P24 strain did not seem to be connected with temperature adaptation because its absence resulted in a haploproficient phenotype at low temperature.

Similarly, absence of the P24 right XIII-subtelomere caused a significant delay in the fermentation kinetics at both 15 and 28 °C, whereas absence of the same region that belonged to P5 significantly improved fermentation at low temperature. The hemizygosity of the left XV-subtelomere was the only one to have an impact on fermentation activity at low temperature. Absence of this region in P5 significantly delayed the end of the fermentation at 15 °C, whereas lack of the P24 region improved this fermentation activity at low temperature.

### Identification of the causative genes of chromosome XIV QTL

**Fig. 6:**
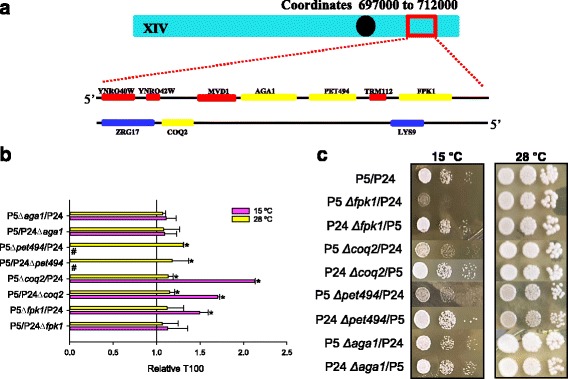
Identification of the causative genes in the QTL of chromosome XIV. **a** Distribution of the genes present in the QTL of chromosome XIV. The genes selected for RHA are colored in *yellow*. **b** Fermentation kinetics (T100) in hemizygous hybrids P5/P24 of genes *AGA1*, *FPK1, COQ2* and *PET494*. The T100 value was compared with the control normalized as value 1. *Significant differences compared with the control at the same temperature. # Indicates stuck fermentation before T100. **c** Spot test between the control hybrid and the hemizygous deletion of the selected genes

The QTL located in chromosome XIV was the only one not to be located in a subtelomeric region. The four genes (*AGA1*, *COQ2*, *FPK1* and *PET494*) closest to the mapped QTL were selected for carrying out reciprocal hemizygosity analyses (Fig. 6a). *MVD1* and *TRM112*, also in this region, were not selected because they are essential genes in the background of BY4741 strains. *AGA1* is an agglutinin involved in sexual reproduction whose deletion has no effect on fermentation activity. However, the hemizygous hybrid strains of the other three genes clearly differed in fermentation activity at low temperature (Fig. 6b).

*PET494* and *COQ2* are mitochondrial proteins whose presence in hemizygosity produces impaired fermentation activity in both strains and at both temperatures, but mainly at a low temperature. *PET494* is a translational activator of one of the subunits (*COX3*) of cytochrome c oxidase [33] and *COQ2* encodes a transferase that catalyzes the second step in ubiquinone (coenzyme Q) biosynthesis [34]. The deletion of both genes provokes the absence of respiratory growth.

Finally, *FPK1* is a Ser/Thr protein kinase that phosphorylates several aminophospholipid translocase family members (flippases) by regulating phospholipid translocation and membrane asymmetry. The reciprocal hemizygote that carries the P5 allele has no effect on the fermentation kinetics at any temperature. However, the hemizygous strain that carries the P24 allele caused a substantial delay (~340 h) in low-temperature fermentation. The impaired fermentation activity of P5 Δ*fpk1*/P24 was also confirmed by a spot assay (Fig. 6c), which also showed an important growth defect of this hemizygous strain at low temperature. P24 has a substitution R520K in this gene that could be the cause of the inferior phenotype of this strain at low temperature.

### RH analysis of wine-lab strain hybrids for the presumptive individual genes contained in the QTL subtelomeric regions

**Fig. 7:**
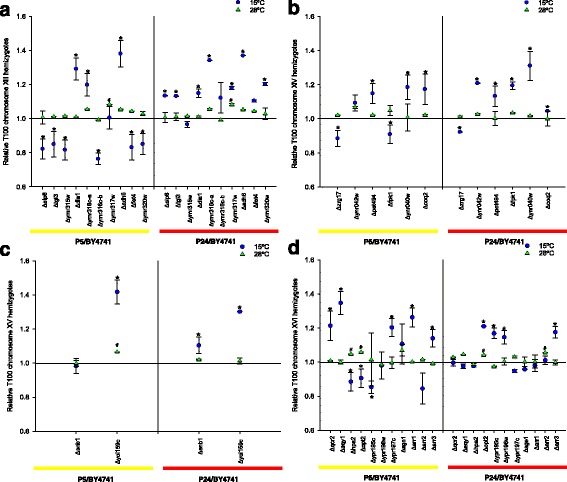
RH analysis of the genes present in the QTL regions of chromosomes XIII (**a**), XIV (**b**), XV (**c**) and XVI (**d**). Hybrids were constructed using the mutants of the BY4741 collection and haploid parental strains. T100 is the time needed to consume the total amount of sugars present in must. The T100 value was compared with the control normalized as value 1. *Significant differences compared with the control at the same temperature

The truncation of subtelomeric regions involved the deletion of some genes not connected with low temperature. As we have information only on the genes present in the first sequenced strain (S288c), in which special efforts were made to clone and sequence each telomere region, we used the sequence available in the *Saccharomyces* Genome Database (SGD: http://www.yeastgenome.org) to perform a large-scale RH analysis with the genes known to be present in subtelomeric regions (~20–30 Kb), and those that are not essential (Additional file 6: Table S2) [35]. The haploid single gene deletion strains in the background of the BY4741 lab strain were crossed with the stable haploids of each parental wine strain by constructing hemizygous hybrid lab/wine strains (BY4741/P5 and BY4741/P24). The fermentation capacity of the resulting hemizygotes, which contained only the wine strain allele of each individual candidate gene, were tested on SM at 15 and 28 °C and compared to the corresponding capacity of the lab/wine hybrid with both parental alleles intact (Fig. 7). This strategy allowed us to compare the fitness of the different wine alleles and to attempt to discriminate the contribution made by each gene to the phenotype.

The RH analysis of individual genes showed both haploinsufficient and haploproficient strains in low-temperature fermentation activity. Haploproficiency meant that only the retention of the wine allele sufficed to improve the fermentation fitness of this hemizygous strain. Most haploproficient strains retained the P5 allele, which reinforces the superior fitness of this strain to grow and ferment at low temperature. Conversely, haploinsufficiency denoted a major function in low-temperature adaptation because a drop in the gene-dose impaired the fitness of the hemizygous strain. Most of these hemizygous strains had either a minimal or null impact on fermentation activity at 28 °C whereas they were severely affected at low temperature. At 15 °C, the hemizygosity in most of the tested genes produced haploinsuffiency in the hybrid of both wine strains. Of the genes analyzed in the XVI-subtelomere region (Fig. 7d), *QCR2*, *AQY1*, *YPR197c* and *ARR1* impaired only the low-temperature fermentation activity of the hemizygous P5/BY4741 strains, while genes *OPT2*, *YPR195c* and *YPR196c* affected this fermentation fitness at 15 °C in the hemizygous P24/BY4741 strains. *QCR2* is also a component of the mitochondrial inner membrane electron transport chain and *AQY1* is a spore-specific aquaporin. Finally, *OPT2* is an oligopeptide transporter with a described role in the maintenance of lipid asymmetry between the inner and outer leaflets of the plasma membrane.

The same RHA analysis was also performed with the genes contained in the nonsubtelomeric QTL detected in chromosome XIV (Fig. 7b). This analysis confirmed the relevance of the *FPK1* allele in the superior fitness of the P5 strain because hemizygous P5/BY4741 showed better fermentation activity, whereas hemizygous P24/BY4741 significantly delayed the end of fermentation. Furthermore, the importance of *PET494* and *COQ2* in low-temperature adaptation was also supported because both hemizygotes showed impaired fermentation activity at 15 °C.

## Discussion

Temperature had a strong effect on many life history traits, including growth, development and reproduction [36]. To elucidate the genetic basis that underlies low-temperature adaptation, we followed a method that began with a hybrid generated by crossing two strains with a divergent phenotype. This hybrid was used to generate segregating populations of a very large size, which were selected at environmental pressure (low temperature). Finally, QTL mapping was performed by identifying the regions of allelic enrichment by sequencing the segregant populations. This strategy has been previously applied to identify the QTLs responsible for heat tolerance in *S. cerevisiae* [9, 37, 38], but not to detect the genetic structure of low-temperature adaptation. Our study has also some particularities in that it makes it different to other similar approaches.

Most QTL analyses use strains that are highly divergent with a high density of segregating sites. Liti et al., (2009) reported the presence of five genetically diverged clean lineages in *S. cerevisiae* (populations that do not interbreed) and Parts et al., (2011) stated that the strains which belonged to clean lineages are ideal for linkage analyses as they have an even distribution of segregating sites across the genome, and these polymorphisms have coevolved in a specific genomic context. However, two strains that belonged to the same lineage were crossed (Wine/European) and were very close phylogenetically, as evidenced by the whole-sequence analysis of both strains. No more than 15% of the SNPs detected in both strains compared with the reference S228c were private mutations. While the low density of segregant sites between both strains proved to be an added difficulty for the QTL analysis since information about neighboring sites could not be used for more accurate allele frequency inferences, the strategy of intercrosses of segregants during the multiple generations that broke up linkage groups allowed individual QTLs to be determined and reduced to small numbers of variants [32, 37].

Another different feature of our study compared with the commonly used bulk segregant analyses was the individual characterization of the 300 F13 segregants at their growth rate at low temperature. Segregants exhibited a wide range of cold tolerance phenotypes, but also a high percentage of these segregants displayed heterosis (hybrid vigor). This result revealed that the arrangement of alleles between both strains produced some combinations that improved fitness for it to become superior to parentals. This suggests that both strains contained alleles which contributed to the cold tolerance phenotype of these transgressive segregants. However, this result was also very interesting for the industrial exploitation of these strains. The intercross during many generations of two industrial strains and the further selection of fitter genomic combinations by growth during several generations under selective pressure is a good genetic improvement method. These improved strains may be rapidly transferred and easily accepted by industry, which rejects the use of GMOs.

Our QTL analysis revealed the importance of the subtelomeric regions that contained a genetic variation responsible for traits of interest. Subtelomeric regions are generally incomplete in most genome projects. However, these regions cannot be ignored because, in yeast, 25% of all QTLs for many assessed traits map to beyond the last markers available. Yet the region contains only 8% of the genome [39]. Subtelomeric gene families evolve faster than their internal counterparts, and subtelomeric regions are more frequently sites of gene duplication [40], which suggests a unique role of subtelomeres as hotbeds for genomic evolution and innovation [41]. The whole-sequence analysis of parental strains also supports the idea that some of this variation might be due to the copy number of the genes present in different subtelomeres [32].

The hemizygotic truncation of one copy of the subtelomere in isogenic hybrid strain P5/P24 had antagonistic effects depending on the parental origin of this region. Thus the P5 right subtelomere of chromosome XVI must contain genes of much importance for wine fermentation, regardless of the temperature of this process. Conversely, the same region in P24 must not comprise these fermentative genes as some gene has a detrimental effect on the fermentation activity at low temperature. However, the most paradigmatic example of genomic regions that confer superior fitness to the P5 strain at low temperature is the left subtelomere of chromosome XV. The fermentation performance of the hemizygous strain that lacked the P5 copy was clearly affected at low temperature, but not at 28 °C, and the hybrid strain that carried the P5 copy in hemizygotes significantly improved. Application of new long read sequencing technologies would reveal genes residing in these subtelomeric regions, and allow identifying the causal genes involved in adaptation at low temperature.

We detected only a nonsubtelomeric QTL in chromosome XIV. In this case, the RH analysis showed two mitochondrial proteins, which are essential for respiratory growth and extremely important in fermentation activity at low temperature. Although we may think that the absence of respiratory metabolism could not be important during a process with a dominant fermentative metabolism, it is well-known that the yeast strains which lack the mitochondrial function are sensitive to oxidative stress caused by reactive oxygen species (ROS) [42]. We recently proved that cells grown at low temperature are also subjected to stronger oxidative stress [27, 43]. The RH analysis also showed that gene *FPK1* was very important for endowing better cold adaptation to P5 strain. This gene regulates flippase activity, which establishes plasma membrane asymmetry by flipping specific phospholipids from the extracellular to the cytosolic leaflet. The continuous remodeling of the phospholipid composition of cellular membranes as a response to low-temperature fermentations has been widely reported [44–47]. The implication of *FPK1* in low-temperature adaptation could be due to an improvement in the ability to maintain plasma membrane asymmetry. Finally, the RH analysis of the BY4741/wine hybrid strains revealed the key role during low-temperature fermentations of a gene (*QCR2*) that encodes a protein of the mitochondrial inner membrane and a gene (*OPT2*) involved in lipid asymmetry maintenance between the inner and outer leaflets of the plasma membrane, among others. This demonstrates the importance of both a fit mitochondria and the capacity of lipid remodeling in the plasma membrane for coping with low temperatures.

## Conclusions

We followed a thorough strategy, previously described by Parts et al. (2011) [37], to elucidate the molecular basis that determines fitness or better adaptation during wine low-temperature fermentations. The QTL analysis and further RH analyses of the detected QTLs proved the importance of subtelomeres as a source of variation in industrial yeast and the need to invest efforts in sequencing these regions with new sequencing technologies with long reads from single molecules. We detected the individual genes involved in adaptation at low temperature fermentation by highlighting the importance of a fit mitochondria, perhaps for coping with greater oxidative stress at low temperature, and the maintenance of correct asymmetry and phospholipid distribution in the plasma membrane. Altogether this information is very useful for industrial yeast exploitation because the mechanisms involved in cold adaptation can be used as important traits for future selections of industrial cryotolerant strains or for their genetic improvement.

## Methods

### Strain selection and advanced intercross lines

We selected two industrial wine strains, P5 and P24, as parent strains for their marked phenotypic growth differences at low temperature [27]. Both strains were kindly provided by Lallemand Inc. (France). We generated derivative strains that were stable haploid and auxotrophic for uracil (ho::KanMX4 *ura3*Δ and ho::NatMX4 *ura3*Δ). The selected haploids didn’t show significant differences (*p*-value ≤0.05) in the growth rate with the parental strains (diploids) both at 28 and 15 °C. We replaced the *LYS2* with *URA3* gene in the P5 strain. Such gene replacement restores the ability of growth in the absence of uracil and makes it unable to grow without lysine. The two parental strains with opposite mating types (P5 *mat α URA3*+ and P24 *mat a LYS2*+) were crossed in complete media (YPD) and grown overnight. Patches were replica-plated in synthetic minimal media (SD) to select diploid F1 hybrids. Two F1 hybrid replicas were grown overnight (spread over a whole Petri dish) and replica-plated on KAc (potassium acetate 1%, agar 2%) at 30 °C to be sporulated for 10 days. Sporulation efficiency (% of sporulating cells) was monitored until it reached >90%. The cells from the whole plate were carefully collected and resuspended in 0.5 mL of sterile water, treated with an equal amount of ether and vortexed for 10 min to selectively kill unsporulated cells [48]. Cells were washed 4 times in sterile water, resuspended in 900 μL of sterile water and treated with 100 μL of zymolase (10 mg mL^−1^) to remove the ascus. Cell mixtures were vortexed for 5 min to increase spore dispersion and inter-ascus mating.

Cells were plated at high density to begin a second round of mating and meiosis (F2). The presence of two distinct markers (*LYS2* and *URA3*) at the same genomic position prevents them from co-segregating in haploid cells, and thus allows selection for diploid cells segregating at that locus. In order to confirm this system, we dissected 10 tetrads from the F6 pool, all of which had the correct 2:2 segregation of the *LYS*/*URA* markers. The sporulated pool was treated with zymolase and plated at high density to start the next intercross generation, as described in the paragraph above (Additional file 7: Figure S5). This process was repeated until the F13 population was achieved to generate a large pool of segregants for sensitive and high resolution QTL mapping [37].

### Media and growth conditions

The growth media selected for the experiments were SD (Yeast Nitrogen Base (YNB, Difco) supplemented with 20 g L^−1^ of glucose as the carbon source), YPD (glucose 20 g L^−1^, peptone 20 g L^−1^, yeast extract 10 g L^−1^) and synthetic grape must (SM), which was derived from that described by Riou et al. (1997) [49]. The SM composition included 200 g L^−1^ of sugars (100 g L^−1^ glucose +100 g L^−1^ fructose), 6 g L^−1^ malic acid, 6 g L^−1^ citric acid, 1.7 g L^−1^ YNB without ammonium and amino acids, anaerobic factors (0.015 g L^−1^ ergosterol, 0.005 g L^−1^ sodium oleate and 0.5 mL L^−1^ Tween 80) and 0.060 g L^−1^ potassium disulfite. The assimilable nitrogen source used was 0.3 g N L^−1^ (0.12 g N L^−1^ as ammonium chloride and 0.18 g N L^−1^ in an amino acid form; the proportion of each amino acid was administered as previously proposed by Riou et al. (1997) [49]. Growth was monitored at 600 nm in a SPECTROstar Omega instrument (BMG Labtech, Offenburg, Germany). Measurements were taken every 30 min for 4 days after 20-s pre-shaking for all the experiments. At low temperatures (15 °C) however, microplates had to be incubated outside the spectrophotometer and then placed inside before being measured (every 3 h for 10 days). Microplate wells were filled with the required volume of inoculum and 0.25 mL of medium to always ensure an initial OD of approximately 0.1 (inoculum level of about 10^6^ cells mL^−1^). Uninoculated wells for each experimental series were also included in the microplate to determine, and to therefore subtract, the noise signal. All the experiments were carried out at least in triplicate. Growth parameters were calculated from each treatment by directly fitting OD measurements versus time to the reparameterized Gompertz equation proposed by Zwietering et al. (1990) [50]:

$$ \mathrm{y}=\mathrm{D}\ast \exp \left\{- \exp \left[\left(\left({\upmu}_{\max}\ast \mathrm{e}\right)/\mathrm{D}\right)\ast \left(\uplambda -\mathrm{t}\right)\right)+1\Big]\right\} $$

where y = ln(OD_t_/OD_0_), OD_0_ is the initial OD and OD_t_ is the OD at time t; D = ln(OD_t_/OD_0_) is the asymptotic maximum, μ_max_ is the maximum specific growth rate (h^−1^), and λ the lag phase period (h).

For the spot assays, the cells were grown on YPD at 28 °C to the stationary phase (OD600~4) were harvested by centrifugation, washed with sterile water, resuspended in sterile water to an OD (600 nm) value of 0.5, and followed by serial dilution. From each dilution, 3.5 μL were spotted onto YPD agar plates. Plates were incubated at 28 and 15 °C for 2 and 9 days, respectively.

### Selection experiment

The pools of the population size of 10–100 million cells (estimated by plating serial dilutions and colony forming units (CFU) counting) were collected from the sporulation media and treated with ether and zymolase, as described above. Spores were grown in complete media (YPD) and synthetic must (SM), and were incubated at either optimum temperature (28 °C) or low temperature (15 °C) until the stationary phase was reached. All the cells were carefully collected and resuspended in distilled water and a small volume (the volume required to inoculate at an optical density (OD) of 0.2) of the expanded culture was transferred to 60 mL of fresh medium. Culture growth was monitored by measuring absorbance at 600 nm every 24 h at 28 °C, and every 48 h at 15 °C. The number of generations was calculated by the equation: *n* = (log N_t_ - log N_0_)/log2, where n is the number of generations, N_0_ is the initial OD and N_t_ is the OD at time t. The experiment was carried out 8 times, after which the selected populations were analyzed.

### Sequencing

The genome sequencing of the selected populations was performed by 5500xl SOLiD sequencing. Genomic libraries were prepared following the manufacturer’s standard instructions. Emulsion PCRs were performed using the SOLiD™ EZ Bead™ Systems. Sequencing was carried out by 75 nt single-end read exact call chemistry (ECC) following the manufacturer’s standard protocols. The whole-genome sequences are deposited in the Sequence Read Archive (SRA) database (http://www.ncbi.nlm.nih.gov/sra/) and are available with access number SRP048919. Breseq (v0.27.1) [51] pipeline was used to first align the reads of the reference S288c genome (using Bowtie2 [52], and to identify SNPs and indels with a frequency cutoff of 0.2. CNV detection was performed using CNV-seq [53] with a window size of 121 nt (with a minimum log2 fold of 0.6 and a minimum *p*-value of 0.001)). P5 was used as the “reference” genome and P24 as the “test genome”. Only CNV larger than 900 bp were considered for the analysis. The sequences of seven informative loci [29] from 15 strains that represented pure groups were downloaded from the SGD (http://www.yeastgenome.org/) and the NCBI (https://www.ncbi.nlm.nih.gov/) to perform the phylogenetic analysis. Each gene sequence was aligned using mafft (v7.221) [54] individually and then concatenated. The phylogenetic tree was constructed using a Maximum Likelihood Method (ML) with RAxML [55] with 100 bootstrap replicates. The distribution of the SNPs among the strains was visualized using Circos 0.69.2 (http://circos.ca).

### Linkage analysis

We retained variants that were covered with at least 30 sequencing reads in both control and selection experiments, and were present at 30–70% allele frequency in the initial segregant population. This ensures that only confident segregating alleles are analyzed. We then calculated the allele frequency changes in both biological replicates for each retained site, and called QTLs the alleles with a frequency change of at least 0.1 in the same direction in both replicates.

### Validation of QTLs

QTLs were validated by reciprocal hemizygosity using the *URA3* gene as a selectable marker [56]. Briefly, the haploid versions of the parental strains (P5 Mat α, ho::HygMX, ura3::KanMX or P24 Mat a, ho::NatMX, ura3::PhleoMX) were used to delete each target subtelomeric region or gene, and to construct all the possible combinations. After the deletions of the candidate regions, strains were crossed to generate the hybrid strains and selected in double drugs plates. Diploid hybrid strains were detected by the benomyl assay [57, 58] and confirmed by Mat locus PCR [59], and the deletions of the target genes were confirmed by PCR using specific primers. The uncharacterized single copy ORF *YMR317W* was used as a target to truncate the chromosome XIII right subtelomeric region, gene *YOL159C* was used to truncate the chromosome XV left subtelomeric region and gene *SGE1* was employed in the case of the chromosome XVI right subtelomeric region. To identify the contribution of the alleles present in these regions, 60 hemizygote hybrids were constructed, each resulting from a cross between a derivative haploid P5 or a P24 strain and BY4741 that lacked one of these genes. A heterozygote hybrid strain with the wild-type BY4741 was also constructed and used as a control.

### Fermentation conditions

Fermentations were performed at 28 and 15 °C, with continuous orbital shaking at 100 rpm. Fermentations were done in laboratory-scale fermenters using 100-mL bottles filled with 60 mL of SM. Fermentations were monitored by the density of media (g L^−1^) using a densitometer (Densito 30PX, Mettler Toledo, Switzerland). Fermentations were considered complete when density reached 995 g L^−1^. Yeast cell growth was determined by absorbance at 600 nm and by plating on YPD.

### Statistical analysis

All the experiments were carried out at least in triplicate. Physiological data were analyzed with the Sigma Plot 12.5 software, and the results were expressed as mean and standard deviation. To evaluate statistical significance, two tailed t-student tests were applied with a *p*-value of 0.05. *P*-values were corrected for multiple testing by the Bonferroni test. Phenotypic data were fitted to the reparameterized Gompertz model by nonlinear least-squares fitting using the Gauss-Newton algorithm as implemented in the nls function in the R statistical software, v.3.0. Phenotype heritability H^2^ was calculated as previously described [60], i.e., H^2^ = ((Var_seg_ − Var_env_)/Var_seg_) × 100, where Var_env_ is the pooled variance among parental measurements and Var_seg_ is the variance among phenotype values for segregants. Transgressive segregation was defined as in [24, 60] by the number of segregants whose phenotype level lay at least 2σ higher than the mean phenotype level of the higher parent, or was 2σ lower than the mean phenotype level of the lower parent. σ was the pooled standard deviation of parents.

## Additional files

Additional file 1: Table S1. Genomic comparison among strains. Single nucleotide polymorphism (SNPs) population distribution. SNPs were classified according to genome localization and change in protein sequence (nonsynonymous variant). (XLSX 5469 kb)
Additional file 2: Figure S1. Distribution of private nonsynonymous SNPs in P5 and P24 compared to S288c. An external circle indicates P24 and an internal circle indicates P5. Homozygous changes are colored in green, while heterozygous changes are marked in red. (PDF 243 kb)
Additional file 3: Figure S2. Workflow of populations’ selection and sequencing. Cells were grown in complete media (YPD) and synthetic must (SM), and were incubated at either optimum temperature (28 °C) or low temperature (15 °C) until the stationary phase was reached. At this time, the volume required to inoculate at an OD of 0.2 was re-inoculated into 60 mL of fresh medium. The experiment was carried out 8 times after which the selected populations were analyzed and sequenced. (PDF 43 kb)
Additional file 4: Figure S3. Hybrid population phenotyping after the selection experiment compared with the unselected F13 population using the opposite temperature to that used during the selection process (nonspecific improvement). The selected population (SP) in the YPD medium (A) and synthetic must (SM) (C) at 15 °C. The selected population (SP) in YPD (B) and SM (D) at 28 °C. Box plot represents μmax distribution in each population and the black bar inside the box represents the mean value. *Significant differences in the SP compared with the F13. (PDF 66 kb)
Additional file 5: Figure S4. QTL analysis for low-temperature adaptation. The figure shows the allele frequency change of the selected pools at YPD 15 °C (purple), SM 15 °C (pink) and SM 28 °C (blue) compared with the unselected population. QTLs are indicated at the corresponding positions with red (YPD 15 °C), green (SM 15 °C) and orange triangles (SM 28 °C). (PDF 70 kb)
Additional file 6: Table S2. List of genes used in the RH analysis with the BY4741 strain that are present in the subtelomeric regions and are not essential. (XLSX 13 kb)
Additional file 7: Figure S5. Outline of the construction of advanced intercross lines. We carried out a strategy that forces yeast cells through multiple rounds of random mating and sporulation to create advanced intercross lines (AILs). This step can improve genetic mapping in two ways: increasing resolution by reducing linkage and unlinking nearby QTLs. (PDF 168 kb)

## Abbreviations

CFU: Colony forming units
CNV: Copy number variation
indels: Insertions and deletions
KAc: Potassium acetate
OD: Optical density
ORF: Open reading frame
QTL: Quantitative trait loci
RHA: Reciprocal hemizygosity analysis
ROS: Reactive oxygen species
SM: Synthetic must
SNP: Single-nucleotide polymorphism
SP: Selected population
YPD: Yeast extract peptone dextrose
