# Supplementary figures and images for: The genetic architecture of low-temperature adaptation in the wine yeast Saccharomyces cerevisiae
Source: BMC Genomics. 2017 Feb 14;18:159. doi: 10.1186/s12864-017-3572-2 (PMC5310122; doi:10.1186/s12864-017-3572-2)

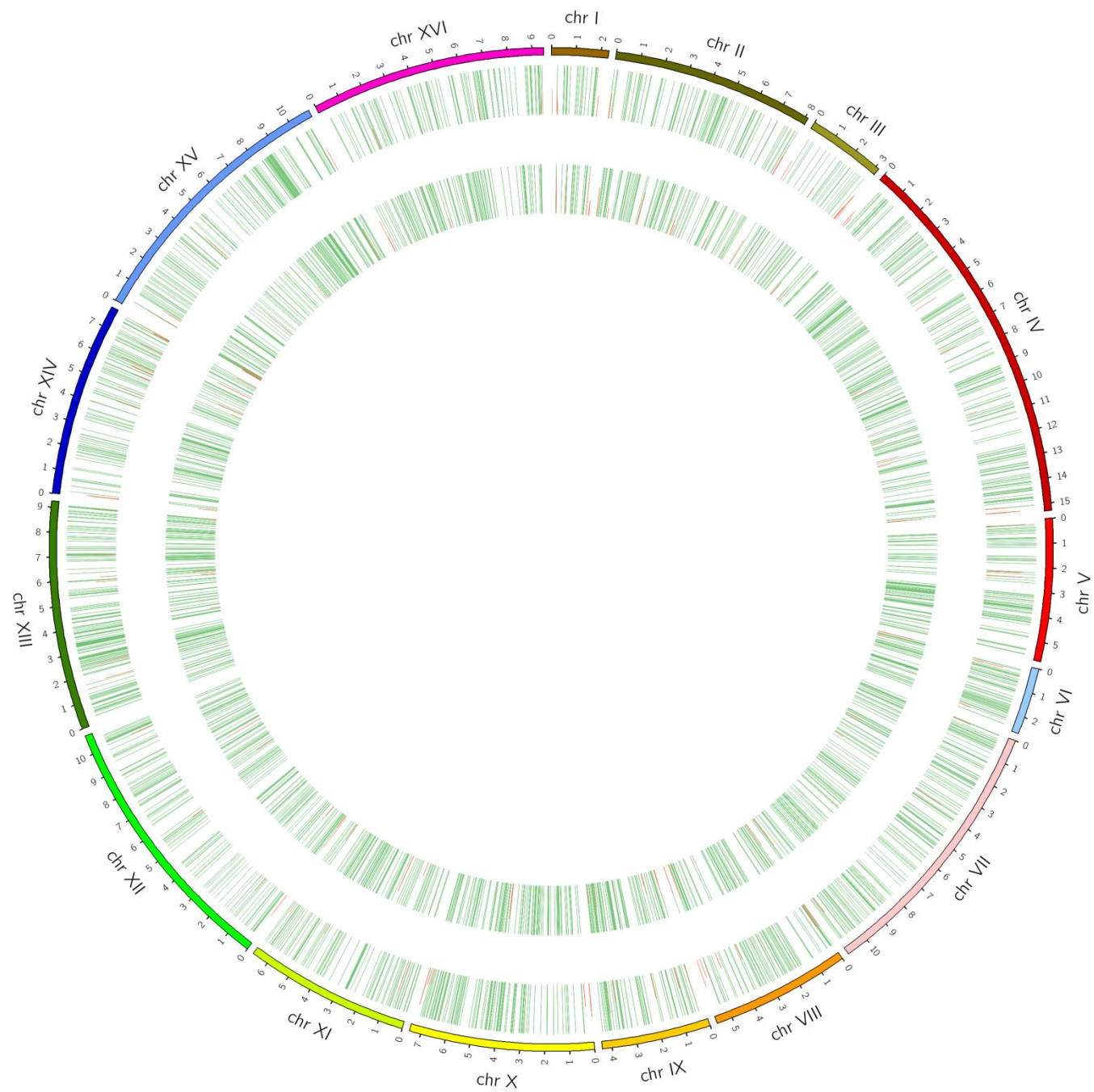

Supplement: Additional file 2: Figure S1. — Distribution of private nonsynonymous SNPs in P5 and P24 compared to S288c. An external circle indicates P24 and an internal circle indicates P5. Homozygous changes are colored in green, while heterozygous changes are marked in red. (PDF 243 kb) [file 12864_2017_3572_MOESM2_ESM.pdf]

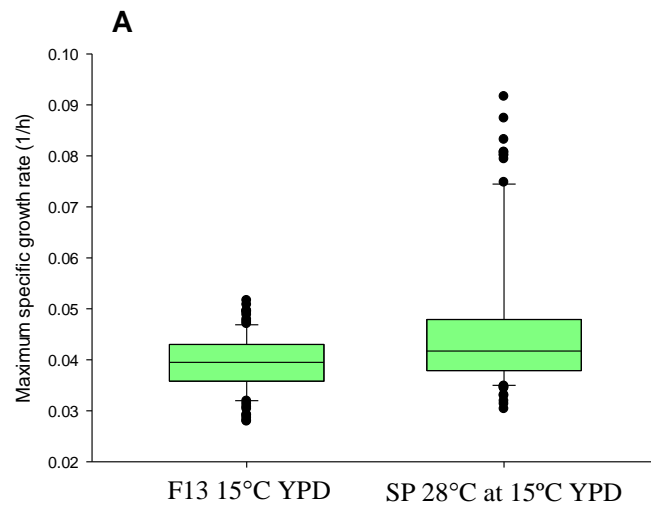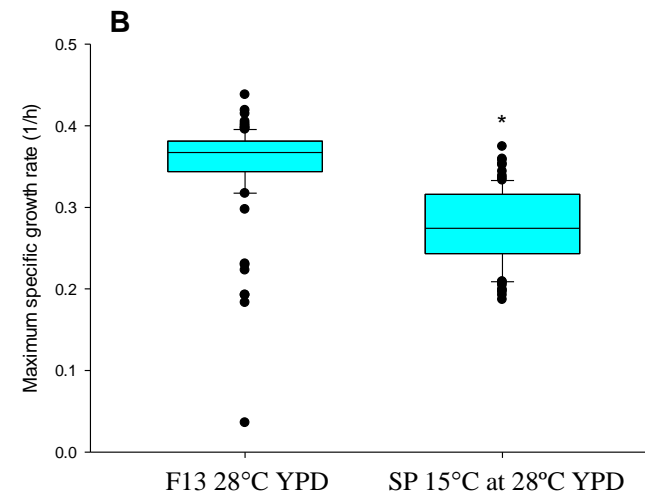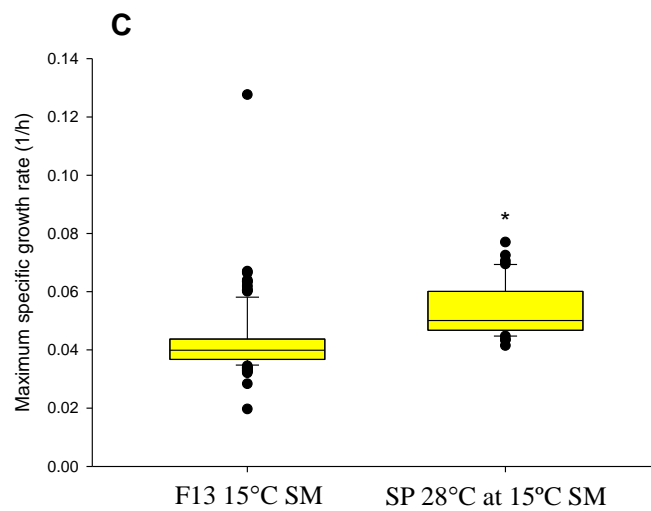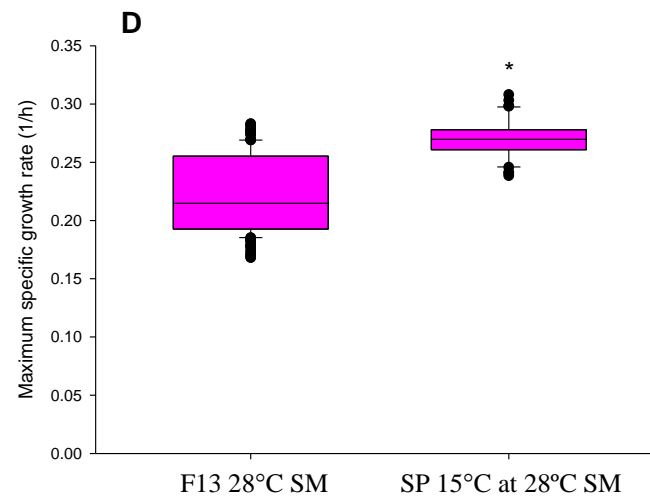

Supplement: Additional file 4: Figure S3. — Hybrid population phenotyping after the selection experiment compared with the unselected F13 population using the opposite temperature to that used during the selection process (nonspecific improvement). The selected population (SP) in the YPD medium (A) and synthetic must (SM) (C) at 15 °C. The selected population (SP) in YPD (B) and SM (D) at 28 °C. Box plot represents μmax distribution in each population and the black bar inside the box represents the mean value. *Significant differences in the SP compared with the F13. (PDF 66 kb) [file 12864_2017_3572_MOESM4_ESM.pdf]

YPD 15 °C

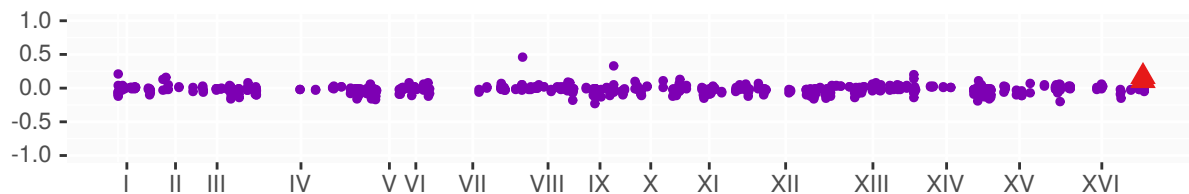

SM 15 °C

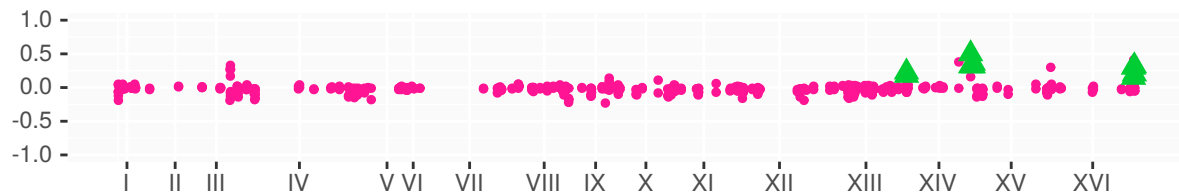

SM 28 °C

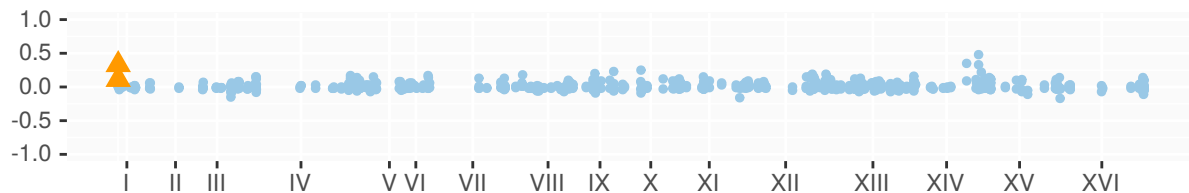

Chromosome

Allele frequency change

Supplement: Additional file 5: Figure S4. — QTL analysis for low-temperature adaptation. The figure shows the allele frequency change of the selected pools at YPD 15 °C (purple), SM 15 °C (pink) and SM 28 °C (blue) compared with the unselected population. QTLs are indicated at the corresponding positions with red (YPD 15 °C), green (SM 15 °C) and orange triangles (SM 28 °C). (PDF 70 kb) [file 12864_2017_3572_MOESM5_ESM.pdf]
