# Supplementary material for: The genetic architecture of low-temperature adaptation in the wine yeast Saccharomyces cerevisiae
Source: BMC Genomics. 2017 Feb 14;18:159. doi: 10.1186/s12864-017-3572-2 (PMC5310122; doi:10.1186/s12864-017-3572-2)
Supplement: Additional file 3: Figure S2. — Workflow of populations’ selection and sequencing. Cells were grown in complete media (YPD) and synthetic must (SM), and were incubated at either optimum temperature (28 °C) or low temperature (15 °C) until the stationary phase was reached. At this time, the volume required to inoculate at an OD of 0.2 was re-inoculated into 60 mL of fresh medium. The experiment was carried out 8 times after which the selected populations were analyzed and sequenced. (PDF 43 kb) [file 12864_2017_3572_MOESM3_ESM.pdf]

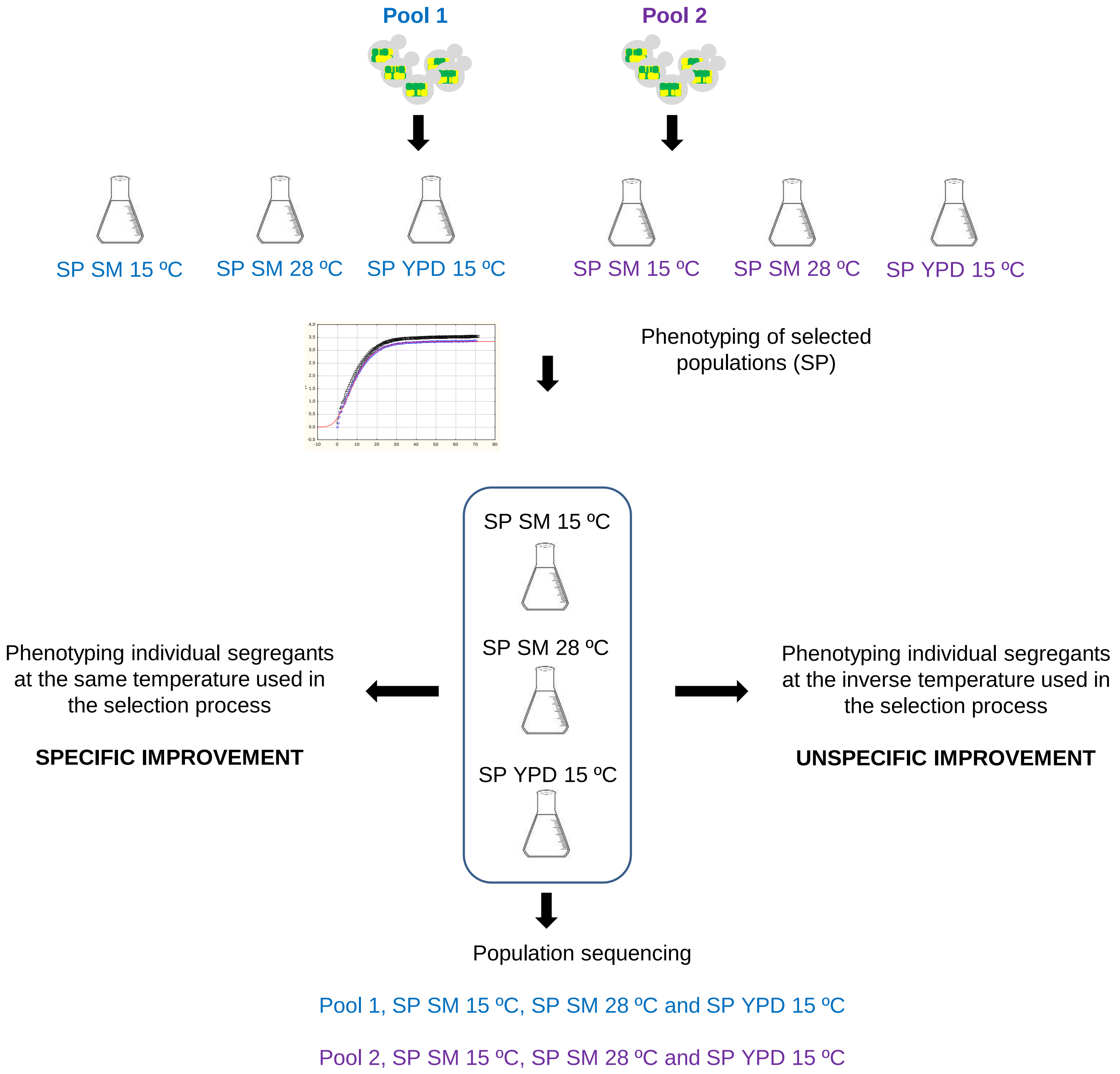
Pool 1
Pool 2
SP SM 15 ºC
SP SM 28 ºC
SP YPD 15 ºC
SP SM 15 ºC
SP SM 28 ºC
SP YPD 15 ºC
Phenotyping of selected populations (SP)
SP SM 15 ºC
SP SM 28 ºC
SP YPD 15 ºC
Phenotyping individual segregants at the same temperature used in the selection process
SPECIFIC IMPROVEMENT
Phenotyping individual segregants at the inverse temperature used in the selection process
UNSPECIFIC IMPROVEMENT
Population sequencing
Pool 1, SP SM 15 ºC, SP SM 28 ºC and SP YPD 15 ºC
Pool 2, SP SM 15 ºC, SP SM 28 ºC and SP YPD 15 ºC
