# Supplementary material for: The genetic architecture of low-temperature adaptation in the wine yeast Saccharomyces cerevisiae
Source: BMC Genomics. 2017 Feb 14;18:159. doi: 10.1186/s12864-017-3572-2 (PMC5310122; doi:10.1186/s12864-017-3572-2)
Supplement: Additional file 7: Figure S5. — Outline of the construction of advanced intercross lines. We carried out a strategy that forces yeast cells through multiple rounds of random mating and sporulation to create advanced intercross lines (AILs). This step can improve genetic mapping in two ways: increasing resolution by reducing linkage and unlinking nearby QTLs. (PDF 168 kb) [file 12864_2017_3572_MOESM7_ESM.pdf]

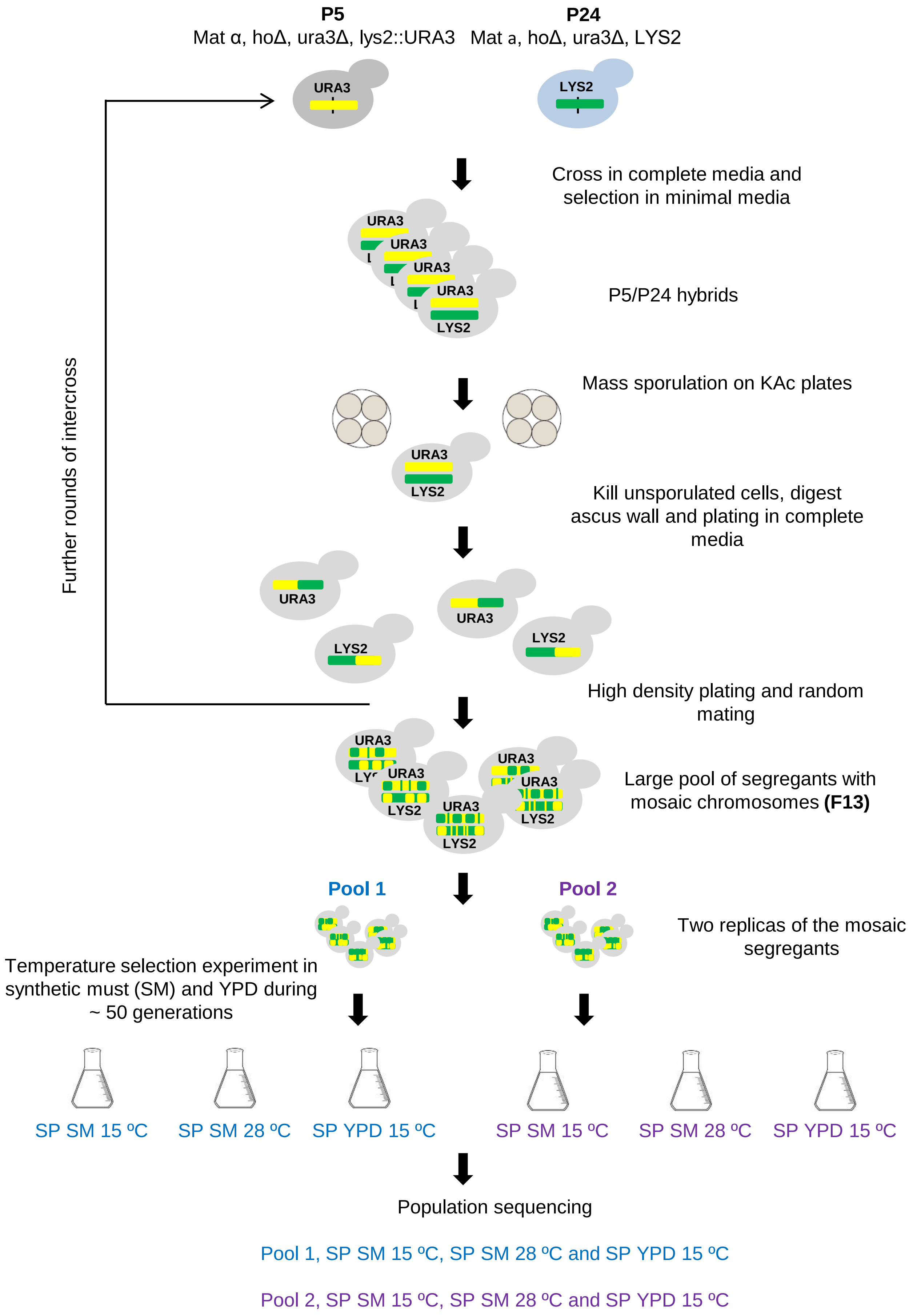
P5
Mat α, hoΔ, ura3Δ, lys2::URA3
P24
Mat a, hoΔ, ura3Δ, LYS2
URA3
LYS2
Cross in complete media and selection in minimal media
P5/P24 hybrids
Mass sporulation on KAc plates
Kill unsporulated cells, digest ascus wall and plating in complete media
High density plating and random mating
Further rounds of intercross
Large pool of segregants with mosaic chromosomes (F13)
Pool 1
Pool 2
Two replicas of the mosaic segregants
Temperature selection experiment in synthetic must (SM) and YPD during ~ 50 generations
SP SM 15 ºC
SP SM 28 ºC
SP YPD 15 ºC
SP SM 15 ºC
SP SM 28 ºC
SP YPD 15 ºC
Population sequencing
Pool 1, SP SM 15 ºC, SP SM 28 ºC and SP YPD 15 ºC
Pool 2, SP SM 15 ºC, SP SM 28 ºC and SP YPD 15 ºC
